# Development of a Generic Microfluidic Device for Simultaneous Detection of Antibodies and Nucleic Acids in Oral Fluids

**DOI:** 10.1155/2013/543294

**Published:** 2013-02-14

**Authors:** Zongyuan Chen, William R. Abrams, Eran Geva, Claudia J. de Dood, Jesús M. González, Hans J. Tanke, R. Sam Niedbala, Peng Zhou, Daniel Malamud, Paul L. A. M. Corstjens

**Affiliations:** ^1^Rheonix, Inc., Ithaca, NY 14850, USA; ^2^Department of Basic Science and Craniofacial Biology, New York University College of Dentistry, New York, NY 10010, USA; ^3^Department of Molecular Cell Biology, Leiden University Medical Center, Building 2, S-01-030, P.O. Box 9600, 2300 RC Leiden, The Netherlands; ^4^Department of Chemistry, Lehigh University, Bethlehem, PA 18015, USA; ^5^Department of Medicine, NYU School of Medicine, New York, NY, USA

## Abstract

A prototype dual-path microfluidic device (Rheonix CARD) capable of performing simultaneously screening (antigen or antibody) and confirmatory (nucleic acid) detection of pathogens is described. The device fully integrates sample processing, antigen or antibody detection, and nucleic acid amplification and detection, demonstrating rapid and inexpensive “sample-to-result” diagnosis with performance comparable to benchtop analysis. For the chip design, a modular approach was followed allowing the optimization of individual steps in the sample processing process. This modular design provides great versatility accommodating different disease targets independently of the production method. In the detection module, a lateral flow (LF) protocol utilizing upconverting phosphor (UCP) reporters was employed. The nucleic acid (NA) module incorporates a generic microtube containing dry reagents. Lateral flow strips and PCR primers determine the target or disease that is diagnosed. Diagnosis of HIV infection was used as a model to investigate the simultaneous detection of both human antibodies against the virus and viral RNA. The serological result is available in less than 30 min, and the confirmation by RNA amplification takes another 60 min. This approach combines a core serological portable diagnostic with a nucleic acid-based confirmatory test.

## 1. Introduction

Infectious diseases including malaria, pulmonary tuberculosis, and viral infections (e.g., human immunodeficiency virus (HIV)) remain major public health problems particularly in the developing world. As individuals unaware of their infection status represent a high risk of transmission, rapid and accurate diagnostics are crucial to decrease incidence and allow for immediate therapeutic intervention. Rapid test devices (RTDs) are available for many infectious diseases allowing appropriate initial screenings. However, these RTDs typically require confirmation by a second test. Currently, confirmatory diagnostics are conducted in well-equipped clinics staffed with trained personnel. Moreover, confirmatory tests generally require a second visit to the clinic, and patients often do not return to collect confirmatory test; this reduces effectiveness in respect to prompt treatment of the infectious disease [1, 2].

Many of the available RTDs used in point-of-care (POC) settings use lateral flow in combination with a visual interpretation of the test result, and these devices do not always exhibit the expected sensitivity and specificity [3]. Often, this performance is a consequence of the testing conditions including how the clinical sample is collected and the level of operator's experience with the test. In resource-limited settings, confirmation of the infection is often carried out with a different RTD. Although algorithms for serial and parallel testing are effective [4], confirmation of an infection by targeting a different analyte with a more sensitive assay is preferable. 

Microfluidic lab-on-a-chip (LOC) devices performing high complexity assays may bring confirmatory testing from the specialized laboratories to the POC setting [5]. Several bench-top technologies have been translated to microfluidic platforms, sometimes by straightforward miniaturization of benchtop assays [6, 7]. In general, miniaturization effectively reduces the overall assay time, which is an important parameter for POC applications. Besides speed and appropriate specificity and sensitivity, successful integration of LOC diagnostics in the POC setting requires dedicated low cost operating devices. 

To demonstrate active/acute infection, on-chip NA amplification methods have been developed based on their versatility, speed, and high sensitivity and specificity [9–11]. Microfluidic devices allowing detection of a single nucleic acid molecule have been developed [12]. However, amplification of submicroliter starting volumes of a target [13, 14] limits the actual sensitivity achievable because existing devices have not been integrated with an NA concentration step. When evaluating the theoretical lower limit of detection (LOD) of the pathogen in a clinical sample, the target concentration required to get the minimum amount of DNA molecules in the amplification compartment must be considered. Although extreme miniaturization of the amplification compartment will reduce the amounts of reagents and consequently the cost of the amplification reaction, it may negatively impact the LOD. In addition, when analyzing clinical samples such as saliva, plasma, urine, or stool, sample preparation steps are generally required in order to achieve maximum sensitivity. Full integration of sample collection, metering, cell lysis, nucleic acid purification, and concentration in a single POC device remains a challenge [9, 15–17].

**Figure 1:**
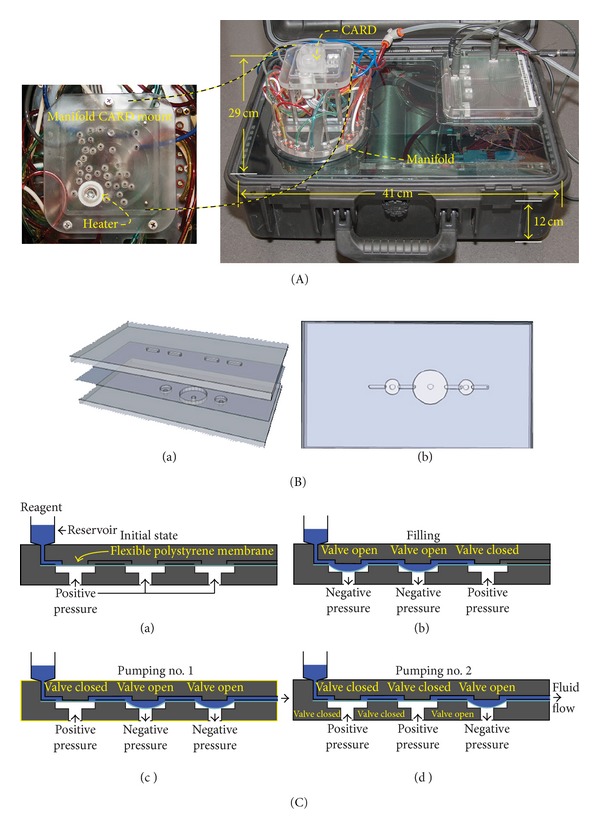
Rheonix processor platform (controller) and CARD technologies. (A) Controller box with integrated vacuum and pressure pump system. A manifold forms the interface between the controller box and the CARD; in the magnified top view the heating module and the solenoid connections (ports) are indicated. Vacuum and pressure ballast tanks are integrated within the controller box. (B (a)) panel showing a minimal schematic of the basic 3-layer PS CARD structure with 2 small and one larger diaphragm (valves/pumps); (B (b)) a top down view of the 3-layer laminated structure shown in panel (B (a)) (without any reservoirs mounted). (C) Diagram showing the valve operations required for peristaltic fluid movement in the CARD.

LOC devices with highly functional modules are particularly useful when they include the capability for multiplexing, allowing for rapid screening and, if necessary, a confirmatory test. Here we describe the development of a device for simultaneous detection of antibody and nucleic acid using a test platform from Rheonix with their previously described CARD technology [18, 19]. The device is a microfluidic CARD designed to receive sample and perform dilution, lysis, NA purification and amplification, and LF-based detection using target-specific LF strips. It can complete the screening and confirmation of an HIV infection within 1 to 2 hours and is easily adapted to detect different targets by changing the NA amplification reagents with the appropriate set of primers and the LF strips with matching capture zones. The device described here can utilize a clinical sample and proceed through the entire “sample-to-result” process automatically. The Rheonix platform employs a portable controller to guide the fluid movement on the microfluidic CARD (Figure 1(A)). The CARD consists of a 3-layer polystyrene (PS) structure with a reagent reservoir layer attached on top (Figure 1(B)). The 3-layer PS structure housing channels and diaphragm valves and pumps represent the core technology of the CARD [18]. The circular chambers beneath valve/pump diaphragms are pneumatically connected to the manifold on the bottom of the CARD. A valve/pump diaphragm is deflected into a chamber when negative pressure is applied which results in the open status of valve/pump. In contrast, when positive pressure is applied to the chamber a valve/pump diaphragm is pushed against the valve seat and forms the closed status of valve/pump. Sequentially actuating multiple diaphragms in series creates a peristaltic pumping action. The capability to fabricate multiple pumps and valves in a single microfluidic chip provides a high degree of flexibility in the manipulation of multiple reagents and specimens, that is, transport of reagents from a reservoir to a reaction chamber, transport of water to a vessel to dissolve dry reagents, active mixing of two or more fluids, discharging of a product to downstream processes, and so forth. To use it, the CARD is first securely mounted with vacuum on the manifold. Microfluidic valves and pumps are pneumatically actuated by 32 individually addressable solenoids controlled by a user-interface program written using script language running on a Java-based software program. The script language program also controls the thermal (cycling) function for nucleic acid amplification, in this case RT-PCR. 

## 2. Materials and Methods

### 2.1. Saliva Samples

Saliva was collected from a healthy volunteer according to protocols described earlier [20]. The UP*link* collector (previously available from OraSure Technologies) and the newly developed collector with solid Porex matrix were tested for potential application in the dual-path CARD. The dual-path CARD was eventually designed for use with the solid Porex matrix collector. Whole mouth saliva samples (WMSSs) clarified using either the collectors or centrifugation (or a combination) were spiked with plasma-based control samples as provided with the OraQuick ADVANCE Rapid HIV-1/2 Antibody Test (OraSure Technologies): a borderline positive and a negative control sample. Samples were furthermore spiked with Armored RNA particles (Asuragen Inc.) as noninfectious replacement for HIV.

### 2.2. RNA Isolation

RNA was isolated using either the High Pure Viral RNA kit (Roche) or the ZR Viral RNA kit (Zymo Research). In the procedures provided with both kits, the lysis/binding buffer could be replaced with Pluronic Lysis Buffer which contained 15% (w/v) of the nonionic detergent heteropolymer Pluronic F-68 (Sigma-Aldrich) in 50 mM Tris-Cl pH 6.6 and 4.5 M Guanidine Hydrochloride. Pluronic lysis buffer can be lyophilized to dryness, stored as a dry reagent, and readily dissolved in H_2_O as required. Proprietary wash buffers from Rheonix with reduced EtOH concentrations were also successfully used to replace the wash buffers provided with the High Pure Viral RNA kit. RNA isolated using the Rheonix wash buffers (either by the benchtop or by on-chip protocols) improved downstream RT-PCR amplification yields. The Roche and Zymo kits utilize spin columns in the RNA isolation; the microfluidic chips were provided with a Rheonix proprietary silica membrane suitable for filtration by vacuum.

### 2.3. Nucleic Acid Amplification

RT-PCR kits were obtained from Roche, Qiagen, GE Healthcare, and Zymo. In most experiments the QIAGEN OneStep RT-PCR Kit (QIAGEN) was used as its hot-start capability allowed convenient on-chip addition of the assay reagents to the microfluidic chip before initiation of the assay. Also, the kit materials could be lyophylized for storage in 0.2 mL microfuge tubes by the proprietary technology of Tetracore Inc., which is a format compatible with the dual CARD NA amplification compartment. The Transcriptor One-Step RT-PCR Kit (Roche) allowed the largest reduction in RT-PCR assay time. The illustra Ready-To-Go RT-PCR Beads (GE Healthcare) were the only commercially readily available dry reagents successfully applied in on-chip RT-PCR amplification. All kits were used to amplify a 155 bp HIV *gag* fragment from RNA isolated from noninfectious Armored RNA particles (Asuragen Inc.). Amplification was performed using primers developed for the COBAS Amplicor HIV-1 Monitor Test v1.5 [21]; the forward (sense) primer was synthesized with a 5′-end Digoxigenin (Dig) hapten and the reverse (antisense) primer with a 5′-end Biotin (Bio) hapten (EuroGenTec).

### 2.4. UCP-LF Detection

Upconverting phosphor (UCP) particles are a highly sensitive reporter suitable for LF format analysis [22], were obtained from OraSure Technologies Inc. (Bethlehem, PA), and were conjugated with 25 *μ*g mouse anti-digoxigenin antibodies or 25 *μ*g protein-A as described [8, 23, 24]. Lateral flow strips for detection of anti-HIV antibodies and Dig-Bio labeled DNA amplicons were also produced as described [8, 23–25] with a 22 mm sample pad. The composition of the high salt lateral flow (HSLF) assay buffer was 270 mM NaCl, 1% w/v BSA (Sigma, A-2153) 0.5% v/v Tween-20 in 100 mM Hepes pH 7.4).

### 2.5. Microfluidic Chip/CARD Technology

Microfluidic chips were provided by Rheonix, Inc. Before designing the entire and comprehensive dual path CARD, various types of simpler intermediate devices were developed to allow the analysis of specific modules and assay steps. Required design changes were indicated by the users and implemented by Rheonix according to methods protocols described in Zhou et al. [18] and Spizz et al. [19].

### 2.6. Operation of the Dual Path Chip

**Figure 2:**
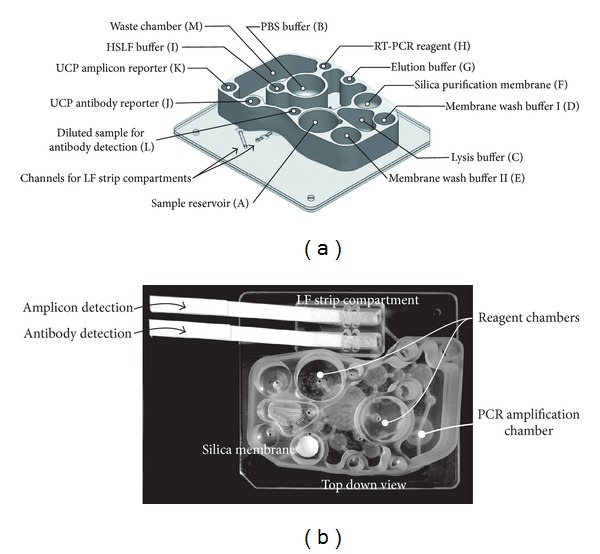
The dual-path antibody RNA CARD. Design (a) and top down image (b) of the dual path microfluidic device with the reagent reservoirs and other compartments used in the detection of anti-HIV antibody and HIV RNA.

**Figure 3:**
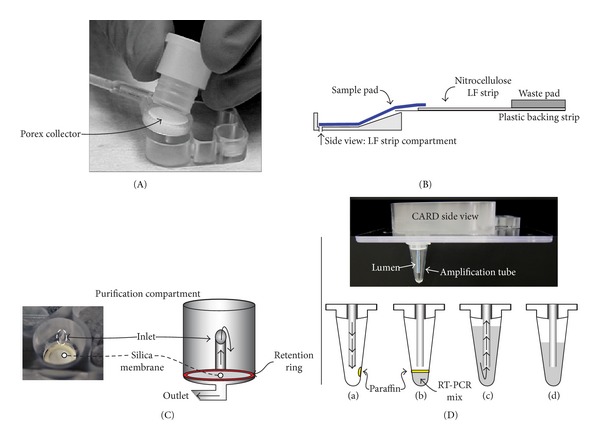
Unique modular components of the CARD. (A) Saliva collector with removable solid Porex matrix. (B) Illustration of the LF strip compartment and the LF strip schematic: the LF strip contains an extended sample pad that is positioned in a trough that prevents overflow of the LF sample pad and strip. (C) Schematic of the compartment with the NA-binding silica membrane; the inlet/outlet of the connected channels are indicated. (D) Schematic of the amplification microtube indicates how the lumen functions as both inlet and outlet. During amplification the opening of the lumen is well above the liquid surface. After amplification, HSLF buffer is added to tube through the lumen, and as a result the opening of the lumen will be below the liquid surface allowing it to function as an outlet.

Saliva from an uninfected individual was used to evaluate the dual path chip. Clarified saliva was spiked with 5% (v/v) of the OraQuick antibody control and 10% (v/v) dilution of Armored RNA (Asuragen Inc.). After buffer and reagents were loaded and 100 *μ*L of saliva added to the sample reservoir, the automated protocol was initiated. Individual on-chip compartments referred to in different steps are indicated in Figure 2 with a few distinctive chip features shown in greater detail in Figure 3. A brief description of the different steps distinguished in the dual path assay protocol is listed in the following.

**Step 1 (loading and dilution of saliva sample)**

200 *μ*L PBS (PBS—Compartment B) is used to release and dilute 100 *μ*L of sample from the oral collector (Sample—Compartment A) by pumping. Note that the use of oral collector with the Porex matrix is shown in Figure 3(A).

**Step 2 (detection of antibody)**

Initiated by transferring a 100 *μ*L aliquot of the PBS diluted saliva sample to Compartment L. Part of the aliquot is further mixed/diluted with HSLF assay buffer from Compartment I while flowing to the antibody detection LF strip (Figure 2(b)). HSLF (50 *μ*L) containing 100 ng UCP-protA conjugate is applied to the strip from Compartment J.

**Step 3 (RNA isolation)**

The remainder (200 *μ*L) of the PBS-diluted saliva sample is mixed with 400 *μ*L of 15% (w/v) Pluronic Lysis Buffer in the Lysis Buffer Compartment (C). The lysate is then drawn through the NA binding silica membrane in Compartment (F). The membrane is washed with 200 *μ*L of Rheonix WB-I (containing EtOH) and twice with 200 *μ*L EtOH free Rheonix WB-II and then air-dried for 5 min. Elution water is pumped (Compartment G) onto the top of the silica membrane and incubated for 1 min. RNA is subsequently eluted and directed through the silica back into Compartment G.

**Step 4 (RT-PCR amplification)**

15 *μ*L RT-PCR reagent is added to RT-PCR reagent Compartment H and pumped to the microtube together with 1 *μ*L of the eluted RNA from Compartment G. A drop of low melting paraffin (melt point <50°C) on the inner wall of the tube (Figure 3(D)) liquefies, covers the reaction mixture, and prevents evaporation during amplification.

**Step 5 (detection of the DIG-BIO labeled amplicons using UCP-CF)**

Immediately after completion of the RT-PCR, 100 *μ*L HSLF assay buffer from Compartment I is transferred to the amplification tube to dilute the RT-PCR reaction mixture. A 10 *μ*L aliquot flows from the amplification tube to the LF strip for NA detection, directly followed by 10 *μ*L HSLF. The DIG-BIO labeled amplicons bound to the Avidin Test line of the DNA detection LF strip are detected with 50 *μ*L of UCP-M*α*DIG conjugate (containing 100 ng UCP particles) from Compartment K.

**Step 6 (scanning of the LF strips and result analysis)**

A dedicated reader is used to read the UCP signal. Results are presented as the ratio of the Test line signal divided by the flow control signal.

## 3. Results

The design of the dual path CARD allowed convenient iterations of the various modules. In our evaluation we distinguish three units: (i) the sample application unit, (ii) the antibody detection unit, and (iii) the nucleic acid unit. Each unit has a modular design with variable complexity. 

### 3.1. Sample Application

In a previous study [26] several commercially available oral collectors were assessed for their use in UCP-based assays. The UP*link* collector (OraSure Technologies) designed to deliver a metered fluid sample directly into a cassette with a LF strip was identified as the most suitable collector. The UP*link* collector was successfully applied in this study, but as this collector is not easily available anymore, an alternative collector was designed. The new collector consists of a circular, solid, porous, and removable Porex disk (Porex Porous Corp., Fairburn, GA; 12.5 mm diameter by 3 mm deep) in a lever and a plastic handlebar. The disk can be conveniently forced out of the lever into a specially designed reagent vessel on the CARD (Figure 3(A)). The current dimension of the Porex collector allows for collection of ~100 *μ*L of oral fluid. After application of the Porex disk to the CARD, the disk was rinsed with two volumes (200 *μ*L) of PBS by pumping fluid reversibly through the disk. The porosity of the disk contributes to the efficient mixing of the saliva sample with PBS, which renders the sample less viscous for subsequent operations. Viscosity issues when analyzing untreated saliva in different types of antibody-only devices are part of a separate study (manuscript in preparation). The diluted sample was split between the antibody and NA analysis pathways.

### 3.2. Antibody Detection

**Figure 4:**
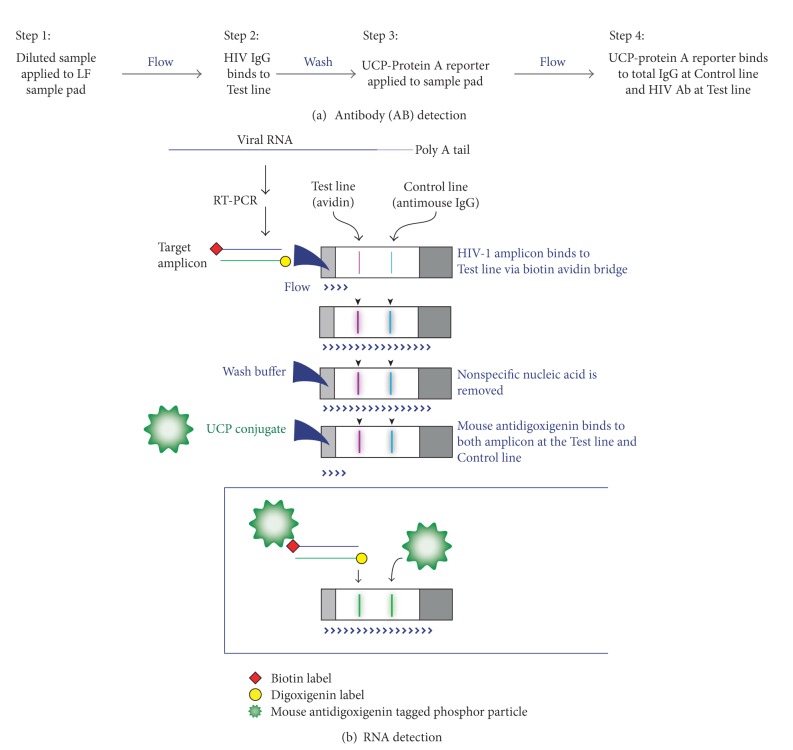
The consecutive flow protocol. (a) Schematic for the analysis of the antibody path, the Test Line was a proprietary peptide mix (OraSure Technologies), and the Flow Line anti-human IgG. (b) Schematic showing consecutive flow applied to detect RT-PCR amplicons provided with a digoxigenin and biotin hapten as described by Corstjens et al. [8], which was developed to detect antibodies against infectious disease pathogens. For analysis of amplicons, the Test line was antidigoxigenin and the Flow Line digoxigenin.

The antibody detection path is directly modeled on the sequential flow assay format (referred to as consecutive flow (CF)) described previously for the detection of antibodies against infectious disease pathogens [8]. The applicability of this assay format for miniaturization was demonstrated earlier with a different type of chip [27]. A schematic of the CF protocol is shown in Figure 4. CF involves three sequential flow steps: the first flow on the LF strip is the clinical sample with the targeted antibody; the second flow is a wash step with HSLF buffer; and the third flow contains UCP reporter particles coated with protein A. The UCP reporter binds to human IgG from the clinical sample at the Test line (T) and Flow Control line (FC); to test samples for HIV infection, T contains HIV-specific peptides and FC contains anti-human IgG. In this study, CF was also used in the NA detection path for the detection of RT-PCR amplicons (Figure 4(b)). It should be noted that in contrast to the approximately vertical placement of the LF strips in tubes (or microtiter plate wells) when performing the benchtop assay, on the CARD they are placed horizontally. Therefore careful regulation of the liquid flow is important to assure a constant fluid stream and to prevent flooding of the nitrocellulose strip. The LF strip holders were designed with a “trough” to allow initiating liquid flow through the extended flexible sample pad of the LF strips without flooding (Figure 3(B)).

### 3.3. Nucleic Acid Unit: RNA Isolation

**Figure 5:**
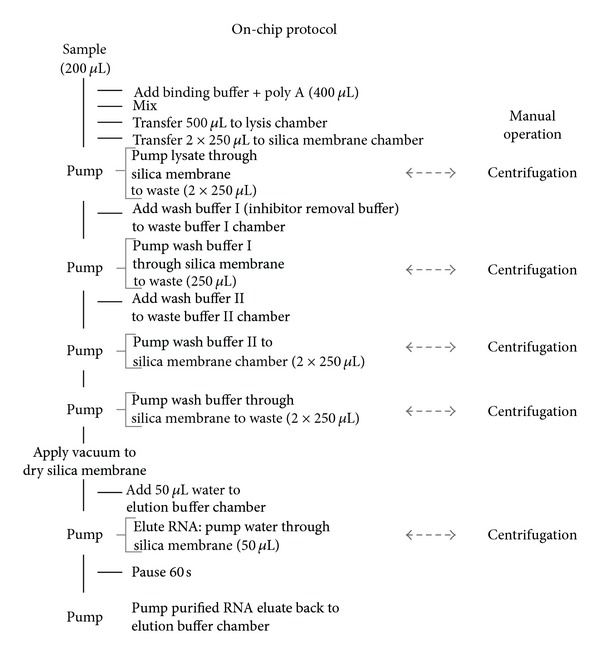
On-chip RNA isolation protocol compared to a typical manual benchtop operation. The bench top RNA isolation protocol involves several centrifugation steps using spin columns provided with a silica purification membrane. For the chip protocol, these steps were replaced by on-chip vacuum filtration.

The NA analysis pathway is the most complex part of the dual path chip. It consists of several modules related to the various procedural steps in the benchtop protocols. The first step is the RNA isolation, by itself consisting of several subprocedures. The conventional protocol to isolate HIV RNA employed the High Pure Viral RNA Isolation Kit (Roche) including spin columns with a silica matrix. Centrifugation steps were replaced with chip-based vacuum filtration (Figure 5). Chips were provided with an RNA isolation compartment fitted with Rheonix proprietary silica membrane. During development the diameter of the matrix was varied between 3 and 5 mm with a constant ~1 mm thickness, and the optimal size was determined to be 4 mm. The larger diameter silica matrices required proportionately greater elution volumes that often resulted in higher concentrations of residual EtOH (or other contaminants) in the eluted RNA and resulted in poor target amplification. Conversely, the smaller sized matrices decreased binding capacity and were easily clogged resulting in slow or blocked flow. Besides the different silica membranes, all other steps in the isolation protocol were carried out identically on both the benchtop and the chip. When required, mixing of fluids was achieved by pumping fluids reciprocally between two reagent compartments. The viscosity of the lysis buffer was of particular concern with respect to efficient mixing and fluid movement and the efficiency of the wash buffers in removing amplification inhibitors. 

#### 3.3.1. Viscosity of the Lysis Buffers

High viscosity buffers represent a problem for fluid transport through narrow channels on a microfluidic chip, and it is especially true for the lysis step. The first action in the on-chip RNA isolation protocol is the transport of diluted sample from the sample reservoir into the prefilled lysis buffer reservoir, which is then reciprocally pumped between compartments until the two fluids are homogenous. The porous matrix used to collect the oral clinical sample (e.g., a Porex disk from the oral collection device (Figure 3) may also facilitate mixing. The concave shape of the bottom of the lysis buffer reservoir assists in the mixing process where sample enters from the bottom into the prefilled lysis buffer reservoir (Figure 3(C)). Lysis buffer from the ZR Viral RNA Kit (Zymo Research) was also successfully used in combination with Rheonix wash buffers. An alternative lysis buffer was developed based on the nonionic detergent heteropolymer Pluronic F-68 and the chaotropic reagent guanidine hydrochloride. Pluronic-based buffers can be lyophilized, stored in dry form, and easily hydrated. The concentration (w/v) of Pluronic reagent in the lysis buffer was optimized and 15% (w/v), was found to be ideal for lysis of viral particles, with high RT-PCR yield and low viscosity. 

#### 3.3.2. Efficiency of the Wash Buffers

**Figure 6:**
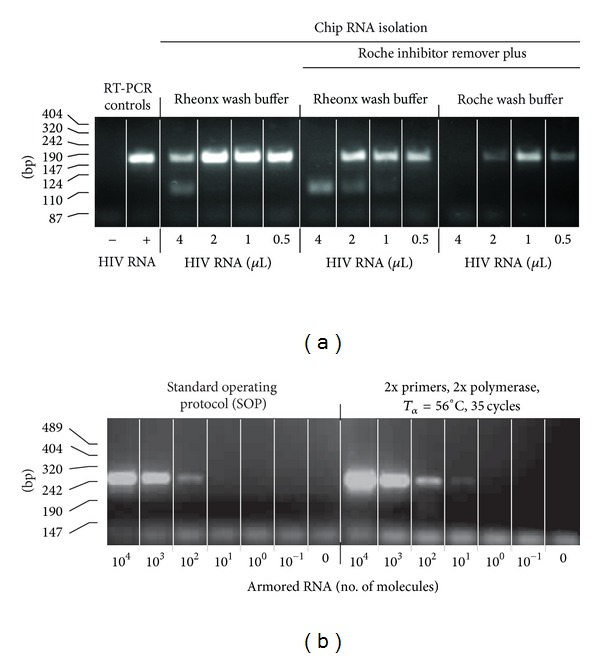
Optimization of the RT-PCR amplicon yield. (a) The effect of different wash buffers on the quality of on-chip RNA isolation was assessed by amplifying increasing amounts of CARD isolated RNA elute by RT-PCR. The volumes represent the amount of eluted RNA used in the amplification reaction using a 10 *μ*L final assay volume. Note the decrease in amplicon yield with increased volume possibly due to the presence of residual EtOH. (b) Doubling of the primers and enzyme concentration and a 2°C lower annealing temperature increased the amplicon yield.

RNA isolated by benchtop and on-chip methods, using reagents from the High Pure Viral RNA Isolation Kit, were both analyzed by benchtop RT-PCR. To prevent variation caused by the on-chip mixing of lysis buffer and sample, mixing of lysis buffer and sample was performed manually before performing chip-based RNA isolation. Nevertheless, a lower yield of RT-PCR product was observed for the on-chip isolated RNA compared to benchtop isolated RNA. Other wash buffers were evaluated for on-chip application, including a proprietary EtOH-free wash buffers from Rheonix (Figure 6(a)). Use of Rheonix wash buffers as an alternative to the EtOH containing High Pure Viral RNA Isolation Kit buffers improved the quality of RNA isolation, which allowed an increase in the amount of eluted RNA in the RT-PCR reaction.

### 3.4. Nucleic Acid Unit: RNA Amplification (RT-PCR)

The second step in the NA pathway is the amplification procedure. Initially PCR-only dedicated chips were used to investigate on-chip RT-PCR conditions for the amplification of a 155 bp *gag* HIV sequence. The amplified region and primers are identical to the Roche Amplicor HIV-1 Monitor test (Roche) version 1.5 [23]. As the various prototype chips used in this research still have an open architecture, Armored RNA (Asuragen Inc.) was used in most cases as a surrogate for infectious HIV for safety reasons. Purified RNA and RT-PCR reagent mix were combined and mixed utilizing the action of the diaphragm valves. Doubling of the enzyme concentration and decreasing the annealing temperature by 2°C improved the amplification (Figure 6(b)). Also, priming (coating) of the channels with mineral oil led to better reproducibility. The optimized method allowed detectable amplification when initiating the RT-PCR with as little as 10 copies of Armored RNA. The potential of using different RT-PCR kits indicates that amplification within a POC functional assay time is feasible. The shortest protocol, using a 5 min RT step, 1 min hot start, and 5 sec each for denaturing, annealing, and extension *per* PCR cycle, was achieved with the Transcriptor One-Step RT-PCR Kit (Roche). For most of the on-chip experiments, the Qiagen OneStep RT-PCR Kit was used since the HotStarTaq DNA Polymerase permitted retaining mixtures of RT-PCR reagents and primers at ambient temperature allowing preloading of the RT-PCR reagents. The use of hot start conditions and polymerases is necessary to limit the formation of primer-dimers and other PCR artifacts when primers and RT-PCR reagents are mixed and preloaded in advance. In the final version of the CARD, dry target-specific amplification reagents will be provided to the amplification compartment, a replaceable 0.2 mL microtube affixed to the bottom of the microfluidic chip. One currently available dry reagent kit that was tested was the Ready-To-Go RT-PCR Beads (illustra, GE Healthcare), This kit performed well on chip using the original protocol and control target (a 425 bp *D. melanogaster* fragment), but it did not accommodate drastic shortening of the cycle time. A complete, ready-to-go reagent cocktail made from reagents supplied with the Qiagen kit and *gag*-specific primers (supplied with a Dig and Bio hapten) was successfully transformed to a dry format by Tetracore Inc., using their proprietary technology [28]; other technologies allowing storage/stabilization of biological at room temperature are available, for example, Biomatrica Inc. (San Diego, CA, USA). The Dig-Bio labeled amplicons were detected using a flow format similar to the one described for the antibody detection (Figure 4(b)), the saliva sample being replaced by a diluted RT-PCR mixture. Lateral flow strips provided with an Avidin capture line and an anti-mouse Flow Control line were used to detect the Dig-Bio amplicons utilizing a UCP reporter coated with mouse anti-DIG antibodies [23].

### 3.5. Performance of the Dual Path CARD to Detect Both Antibody and RNA

A typical experiment with the dual path CARD involves the simultaneous detection of antibody and RNA utilizing saliva containing HIV Armored RNA samples and antibody standards from the OraQuick ADVANCE Rapid HIV1/2 Antibody Test. The open structure allowed convenient manual addition of wet reagents and permitted visual observation of the on-chip fluid transport. In the on-chip protocol, the antibody detection path and the NA detection path proceed sequentially. Buffers and other reagents are loaded into their reservoirs prior to initiating computer control. Once initiated, the fully automated chip-based protocol dilutes the saliva sample with PBS and then transfers an aliquot to the antibody detection path for analysis. This aliquot is further diluted with HSLF assay buffer and transferred to the antibody LF strip containing a test zone to bind HIV-specific antibodies. The sample flow is followed by a wash and a flow of the IgG generic UCP-protA reporter conjugated to detect bound antibody. The strip is dried while the NA analysis path proceeds. NA is extracted from the remainder of the PBS diluted saliva sample by first mixing with Pluronic lysis buffer and then running the lysed sample over the NA-binding silica membrane. The membrane is flushed with the proprietary Rheonix wash buffers and air-dried to help remove residual EtOH prior to elution with nuclease-free water. RT-PCR reagents and eluted RNA are then added to the microtube through a plastic lumen (Figure 3(D)), and the amplification cycle is initiated. The targeted disease/pathogen-specific NA sequence is amplified, and resulting NA amplicons are provided with Dig-Bio tagged primers. Upon completion, HSLF assay buffer flows into the PCR tube through the lumen to mix and dilute the DIG-BIO tagged amplicon; subsequently, an aliquot is transferred to the NA LF strip. The NA LF strip contains an avidin capture line that binds the BIO tagged amplicon. Bound amplicons are then detected by UCP-M*α*DIG reporter particles that are bound to the DIG tag. When LF is completed, both the antibody and NA strips are removed from the CARD and scanned for the presence of UCP label. In future versions the controller box may contain a built-in IR scanner.

**Figure 7:**
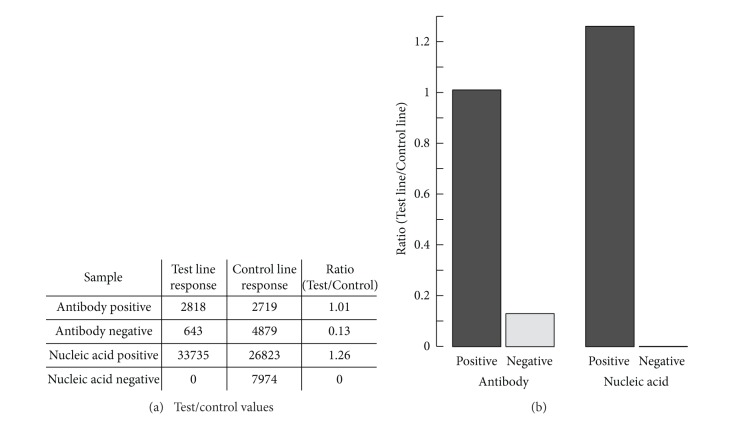
Analysis of saliva samples spiked with HIV RNA as Armored RNA and HIV antibodies on the dual path CARD. (a) Signals representing peak areas (emission in RFU after excitation with 980 nm IR light) of the Test and Flow Control lines. (b) Results are presented as Ratio Values calculated by dividing Test and Control line signals. Ratio values improve the interassay comparison obtained with different LF strips.

The dual path chip was tested with saliva samples spiked with the OraQuick positive control and Armored RNA or with the OraQuick negative control. Chips were loaded with 100 *μ*L of the spiked saliva and run without operator interference.  Figure 7 shows the result obtained after reading the LF strips and indicates a clear distinction between the seropositive and seronegative samples. Analysis of the LF strips gave Test line signals of 2818 relative fluorescent units (RFUs) versus 643 RFU for the low positive and the negative controls, respectively. If required, the assay components/conditions can be adapted in such a way that the negative control does not generate a signal. Above a certain threshold the ratio values are indicative of infection. The threshold is assay and device-specific, and the actual value is determined from a statistically relevant large set of negative controls. When testing patients in a POC setting, the serological results will already be available while the NA path is still in process. In cases where the antibody result indicates infection based on seroconversion, the NA result is required to confirm infection based on the presence of viral RNA. The presence of Armored RNA was also clearly demonstrated; the saliva sample spiked with Armored RNA demonstrated a clear signal (33735 RFU) whereas the control does not result in a signal at the Test line. Ratio values calculated by dividing T and FC signals indicate the same result. RT-PCR results were validated through various RT-PCR benchtop controls using the eluted RNA remaining in Compartment G. 

## 4. Conclusion

This study describes a portable processing system (Rheonix, Inc.) for disposable microfluidic chips suitable for POC applications in the diagnosis, detection, and confirmation of infectious disease pathogens. Prototype devices were developed and assessed for their potential to analyze saliva samples. Each step of the process was evaluated independently, from collection to detection of targets specific to pathogen infection. We note here that besides oral-based fluids, the system was successfully tested with blood-derived samples (mainly plasma and serum) spiked with cultures of HIV (instead of the noncontagious Armored RNA alternative) and can be adapted for use of other body fluids as well. For the oral-based sample analysis the use of a dedicated saliva collector was implemented. The device includes oral fluid-based sample preparation steps required for NA purification and allows the integration of essentially any NA amplification method. The device is suitable for simultaneous detection of multiple types of biomolecules. Here, the detection of antibody and NA is described. Diagnosis of HIV infection was used as a model to screen for the infection through detection of anti-HIV antibodies and confirmation of the actual presence of the virus by detecting an HIV-specific RNA target. This combination of a diagnostic screening with a confirmatory test has major advantages: it allows immediate initiation of therapy and counseling and removes the need for the patient to return to the POC facility for either subsequent testing or a final test result. In addition for populations at high risk for HIV infection, it also provides a method to decrease the window between infection and seroconversion, which is a relatively short period when viral loads are highest and transmission (infection of others) is most likely. Other applications could apply to the detection of anti-HPV (subtype 16 and 18) antibodies [29, 30] and HPV subtype-specific DNA [31–33] in oral fluid as a potential indicator for oral cancer. Also, the prospect of a higher complexity device that can combine the detection of antibodies against helmintic *Schistosoma* species, pathogen-derived NA targets and specific glycosylated-proteinaceous antigens can be explored [34–36]. 

The microfluidic chips are produced by a patented technology (CARD), which is adapted readily for both large- and small-scale production and provides a convenient strategy for rapid modification and testing of small batches of chips for research and development purposes. Different modules were tested and ultimately combined into a single microfluidic chip design for simultaneous detection of nucleic acid (HIV-RNA) and protein (anti-HIV antibody). Alternative methods and modules for several of the on-CARD assay steps described here have been explored (data not shown) and can be integrated depending on the constraints defined for the performance of particular assays. Nucleic acids are detected after amplification; in the example discussed here, RT-PCR was used to amplify an RNA target from HIV. RT-PCR amplification requires a relatively complex heat cycling system; thus we submit that a system capable of performing RT-PCR can be easily adapted for other simpler amplification technologies. To demonstrate this, the system was also successfully tested with a recently commercialized isothermal amplification technology LAMP (results not shown). The only constraint when considering a device with dry reagents is the requirement that the enzymes and other reagents required for amplification need to be provided with a sufficient stability. The dry reagents tested in this study were obtained from a commercial source (GE Health Care) or were dried using a custom proprietary technology (Tetracore Inc.). The assay-specific reagents for the amplification of the RNA target including the target-specific primers can be supplied to the chip in a microtube attached to the bottom of the CARD. In this open architecture research prototype the tubes are easily installed by the operator. The final design will be a disposable and sealed chip that will be discarded after use as biohazard waste. However, the possibility to change the PCR tube with specific amplification reagents remains and adds flexibility with minimal complexity to the system. For nucleic acid detection the processing of the chip will differ only in the technical details required for the specific method of amplification. Amplicons labeled with digoxigenin (DIG) and biotin (BIO) haptens during amplification can be detected by the rapid LF-based method using phosphorescent upconverting phosphor reporters. Required capture zones and dry UCP reporter can be fully integrated in the LF strips which also can be added to the CARD at a later time point. Multiplexing at the NA level [37] can be implemented in LF format by adding different haptens to the amplification reagent mix [26]. Integration of magnetic beads as an alternative to the silica membrane isolation of nucleic acids has been developed and tested (results not shown) and may improve sensitivity since it allows omitting EtOH-based wash steps [38] and NA elution from the protocol with direct input of all bound NA targets in a microliter size amplification reaction. Moreover, magnetic beads may also be applied to capture and concentrate targets other than NA for multiplex analysis. Important to realize is that the modular approach that can be used in the development of specific CARD microfluidic chips in combination with a scalable production facility conveniently allows iteration and optimization of various assay steps in the process of miniaturization. The model used here to explore the Rheonix system and CARD technology is relevant for rapid POC applications to diagnose and immediately validate HIV infections. Robustness, reproducibility, sensitivity, and specificity issues of the current device require further validation with relevant sets of clinical samples using future closed CARD systems. Especially when moving towards applications that include monitoring of the disease (demanding quantitative determination of low copy numbers of HIV viral RNA), aerosol or other potential contamination sources need to be avoided. When focusing on validation of HIV infection in seroconverted patients prior to drug treatment, the constraints regarding HIV RNA quantitation are considerably less and can be clearly achieved with current device.

## Supplementary Material

Supplementary Movie Legend: The operation of an open-structure prototype of the dual-path microfluidic device is shown. The chip was designed for simultaneous detection of antibodies and nucleic acid. The reagent chambers are pre-filled with colored solutions as surrogates for the various reagents and buffers in order to observe the fluid movement and mixing process. Automated processing is initiated after the manual addition of the sample to be analyzed. The antibody detection and the nucleic acid analysis paths proceed sequentially. Note: The timeline for the movie is compressed for demonstration purposes only.

## Abbreviations

Ab: Antibody
BIO: Biotin
CARD: Chemistry and reagent device (a registered trademark of Rheonix, Inc.)
CF: Consecutive flow
DIG: Digoxigenin
HSLF: High salt lateral flow buffer
LF: Lateral flow
LOC: Lab on a chip
LOD: Limit of detection
NA: Nucleic acid
POC: Point of care
RT-PCR: Reverse transcriptase polymerase chain reaction
RTD: Rapid test device
UCP: Up converting phosphor
Up*link*: a registered trademark of OraSure Technologies Inc.
WMSS: Whole mouth stimulated saliva
RFU: Relative fluorescence units.
